# A novel traction method using a multi-loop traction device in colorectal endoscopic submucosal dissection: Anchor traction method

**DOI:** 10.1055/a-2408-9787

**Published:** 2024-09-19

**Authors:** Keisaku Yamada, Masahiro Tajika, Tsutomu Tanaka, Nobuhito Ito, Akihiro Takagi, Yasumasa Niwa

**Affiliations:** 1 Department of Endoscopy, Aichi Cancer Center Hospital, Nagoya, Japan

Colorectal endoscopic submucosal dissection (ESD) continues to present technical challenges. However, several reports indicate that traction devices can reduce procedure time and complications associated with colorectal ESD 1 2 .

Conventional traction devices are limited in their ability to provide traction for large lesions as they can only do so at a single location. Here, we introduce a novel traction method utilizing the commercially available multi-loop traction device (Boston Scientific Co. Ltd., Tokyo, Japan).

A 61-year-old man presented with a 30-mm 0-Is+IIa lesion at the rectum, suspected to be an intramucosal carcinoma ( Fig. 1 ). He underwent ESD ( Video 1 ). Initially, a full circumferential incision was made using an ORISE ProKnife (Boston Scientific). Subsequently, the middle loop of the multi-loop traction device was attached to the reopenable clip (SureClip; MicroTech, Nanjing, China) using threads. The clip, along with the device, was initially attached to the anal side of the lesion. Then, the right loop was attached using a clip, this time on the right side of the lesion. The opposite loop was subsequently attached to the left side of the lesion. Finally, the middle loop was grasped and pulled into the intestinal mucosa on the opposite side of the lesion, allowing for traction in three locations using a single device ( Fig. 2 ).

**Fig. 1:**
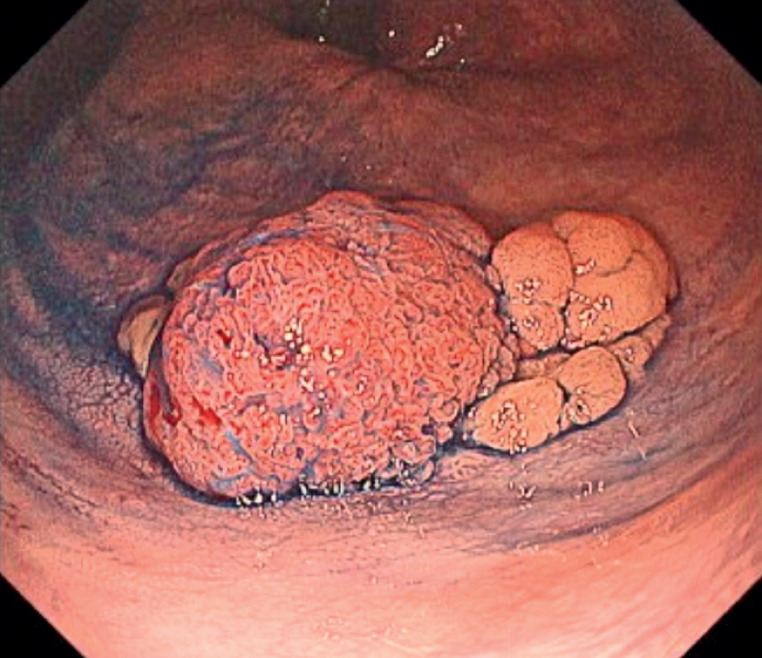
The lesion was a 30-mm 0-Is+IIa lesion at the rectum.

**Fig. 2:**
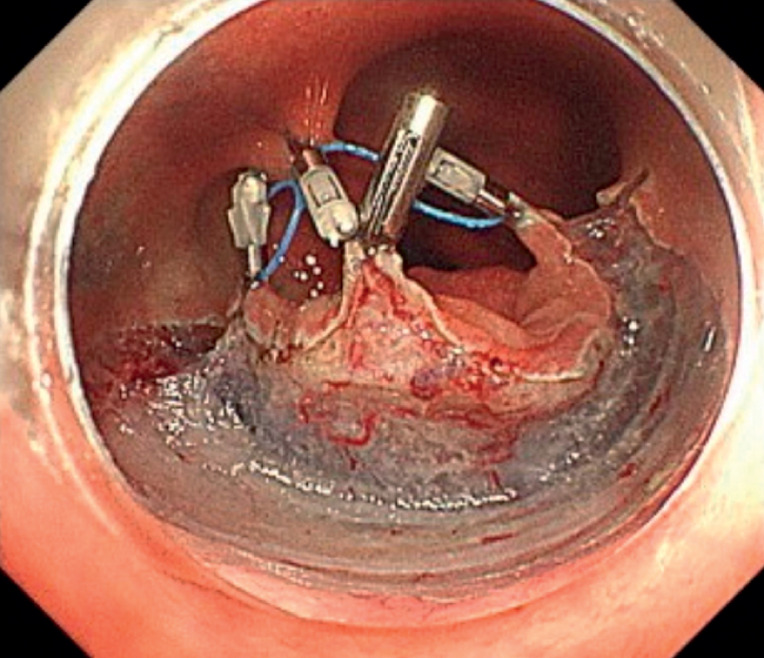
The anchor traction method, which allows multiple traction points, improved the visibility of the submucosal layer.

The anchor traction method allows traction at multiple locations with a single device.Video 1

After applying traction at multiple points, the submucosal layer became clearly visible, enabling the safe performance of ESD using a knife. Pathological analysis revealed that the lesion was a 30 × 25-mm adenocarcinoma in adenoma with negative margins ( Fig. 3 ).

**Fig. 3:**
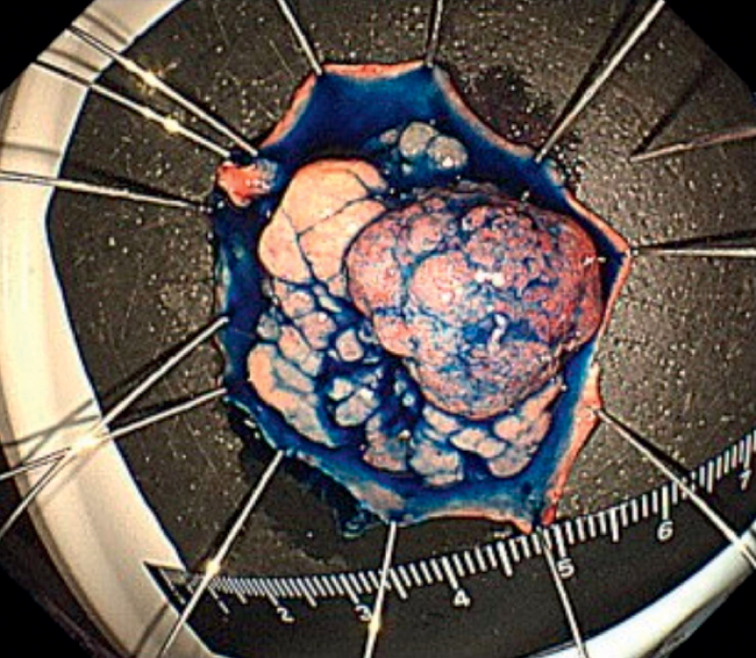
Histopathology showed that the lesion was adenocarcinoma in adenoma with a negative margin.

This novel traction method has been developed to enable traction at multiple locations using a single device, addressing the limitations of the conventional method, and we call it the anchor traction method because the traction shape resembles that of an anchor. The anchor traction method is a safe and effective method in colorectal ESD.

Endoscopy_UCTN_Code_TTT_1AQ_2AD_3AD

## Notes

Endoscopy E-Videos https://eref.thieme.de/e-videos
*E-Videos* is an open access online section of the journal *Endoscopy* , reporting on interesting cases and new techniques in gastroenterological endoscopy. All papers include a high-quality video and are published with a Creative Commons CC-BY license. Endoscopy E-Videos qualify for HINARI discounts and waivers and eligibility is automatically checked during the submission process. We grant 100% waivers to articles whose corresponding authors are based in Group A countries and 50% waivers to those who are based in Group B countries as classified by Research4Life (see: https://www.research4life.org/access/eligibility/ ). This section has its own submission website at https://mc.manuscriptcentral.com/e-videos .
